# Per Se Driving Under the Influence of Cannabis Statutes and Blood Delta-9-Tetrahydrocannabinol Concentrations following Short-Term Cannabis Abstinence

**DOI:** 10.1093/clinchem/hvaf121

**Published:** 2025-11-12

**Authors:** Robert L Fitzgerald, Anya Umlauf, Raymond T Suhandynata, David J Grelotti, Marilyn A Huestis, Kyle F Mastropietro, Igor Grant, Thomas D Marcotte

**Affiliations:** Department of Pathology, University of California-San Diego, San Diego, CA, United States; Department of Psychiatry, Center for Medicinal Cannabis Research, University of California-San Diego, San Diego, CA, United States; Department of Pathology, University of California-San Diego, San Diego, CA, United States; Department of Psychiatry, Center for Medicinal Cannabis Research, University of California-San Diego, San Diego, CA, United States; Institute for Emerging Health Professions, Thomas Jefferson University, Philadelphia, PA, United States; Joint Doctoral Program in Clinical Psychology, San Diego State University/University of California, San Diego, San Diego, CA, United States; Department of Psychiatry, Center for Medicinal Cannabis Research, University of California-San Diego, San Diego, CA, United States; Department of Psychiatry, Center for Medicinal Cannabis Research, University of California-San Diego, San Diego, CA, United States

## Abstract

**Background:**

Several US states have per se laws using 2 or 5 ng/mL of delta-9-tetrahydrocannabinol (THC) as cutpoints for driving under the influence of cannabis, while some have zero-tolerance statutes. These cutpoints are considered prima facia evidence of driving impairment.

**Methods:**

In a cohort of people who regularly use cannabis (*N* = 190) we measured baseline concentrations of THC after instructing participants to abstain from cannabis for at least 48 hours. Baseline driving performance was evaluated using a driving simulator. We also measured blood THC concentrations serially following a smoking session (placebo or active cannabis).

**Results:**

Forty-three percent of the participants exceeded zero-tolerance statutes (≥0.5 ng/mL) at baseline. Twenty-four percent had baseline blood THC concentrations that were ≥2 ng/mL and 5.3% were ≥5 ng/mL. The maximum observed baseline blood concentration was 16.2 ng/mL. Six hours after smoking active cannabis, the median (interquartile range) difference in THC concentrations compared with baseline was 0.5 (0–0.9) ng/mL; a 1-sample *t*-test comparing the mean change to 0 was significant (*P* < 0.001). There was no difference when comparing the mean change to 0 in the placebo group (*P* = 0.69). Simulated driving performance was not different between those who exceed zero tolerance and per se cutpoints vs those who are below these cutpoints (*P* > 0.05).

**Conclusions:**

Many regular users of cannabis exceed zero tolerance and per se THC cutpoint concentrations days after their last use, risking legal consequences despite no evidence of impairment.

## Introduction

Cannabis as a risk factor for causing motor vehicle crashes has been studied in a variety of settings including controlled laboratory studies, at the roadside, and in prospective evaluations (1, 2). Using a driving simulator, our previous work demonstrated significantly worse driving at 30 and 90 min after smoking cannabis ad libitium that recovered to baseline at 4.5 h (3). In a study involving 3000 crashes, Lacey et al. evaluated the contribution of delta-9-tetrahydrocannabinol (THC) on motor vehicle crashes and concluded that the adjusted odds ratio of THC increasing the risk of a crash was 1.0, indicating no increase of crash risk due to detectable THC (4). Brubacher et al. showed that after Canada legalized cannabis there was an increase in the prevalence of drivers who had detectable cannabis in their blood following a motor vehicle crash (5). These authors also examined whether blood THC concentrations were a risk factor for causing crashes (6). They showed a significant association with crash risk for ethanol and when ethanol was combined with cannabis, but were not able to demonstrate a statistically significant increase in crash risk when THC was detected even at concentrations that exceeded 5 ng/mL. Epidemiologic studies show that the crash risk associated with cannabis use is significantly elevated with an odds ratio of 1.32 (7). This compares with an odds ratio of 13 for being involved in a fatal motor vehicle crash when drivers have 0.08% w/v ethanol in their blood (8). A recent meta-analysis of 31 published studies concludes that low-certainty evidence suggests cannabis consumption may be associated with an increased risk of injury due to motor vehicle collisions (2). While all of these approaches listed have strengths and weaknesses, most of the evidence suggests that THC can be impairing but not to the same degree as ethanol intoxication. Developing scientifically valid measures of cannabis impairment is important for maintaining safe roadways.

One approach to regulating driving under the influence of cannabis is to model approaches used for drunk driving. Despite evidence showing no correlation between THC concentrations in biological fluids and driving impairment (3, 9–11), several of the states in the United States have adopted “per se” THC concentration statutes. Other countries have adopted similar approaches (12). In this context, when a driver has concentrations of THC exceeding the per se limits, no additional evidence is required to establish impairment, known as prima facia evidence. In addition to the 6 states that have per se limits, an additional 12 states have a zero-tolerance law (13). The 0.08% w/v per se statue is commonly used for driving under the influence of alcohol, where many reports show that blood ethanol concentrations have a relationship with impairment (4, 14, 15). Even with alcohol, it is recognized that per se limits are not solely based on scientific data but are influenced by legal and societal norms (16). One of the primary problems with using THC concentrations in per se legislation is that the pharmacokinetics of THC are much different from ethanol. Ethanol concentrations are typically undetectable 24–48 h after last exposure, while THC can be measured up to 30 days after last use (17). The duration of THC impairment depends on many factors including the route of administration, and how much was consumed. After inhalation, the effects of THC typically last about 3–5 h, while orally administered THC effects can last for 8 h (18).

This paper reports on baseline concentrations of THC (and related cannabinoids) in people who regularly use cannabis (*N* = 190) and were asked to abstain from using it for at least 48 h prior to phlebotomy. We examined baseline concentrations prior to participants smoking cannabis and compared baseline concentrations with those at the end of the study protocol, 5 h after smoking cannabis. We also report on simulated driving performance for participants who exceeded per se cutpoints compared with participants who were below these values.

## Materials and Methods

All aspects of this human research were conducted in accordance with the Declaration of Helsinki. Full details of inclusion and exclusion criteria have been published previously (3). Briefly, study participants were required to be active users of cannabis (using cannabis 4 or more times in the past month), aged 21–55 years, hold a valid driver’s license, and drive at least 1000 miles in the last year. Exclusion criteria included: a positive urine screen for nonprescription amphetamines, benzodiazepines, barbiturates, opiates, oxycodone, cocaine metabolite, or phencyclidine. To help ensure that participants did not use cannabis at home prior to the study day we performed an oral fluid screen and excluded anyone with an oral fluid THC concentration that exceeded 5 ng/mL.

We measured baseline concentrations of THC in blood after instructing participants to abstain from cannabis use for at least 48 h prior to the study. THC was quantified in whole blood using isotope dilution liquid chromatography with tandem mass spectrometry (LC-MS/MS) as described previously (19). The lower limit of quantification for THC was 0.5 ng/mL. Zero-tolerance statutes refer to any detectable THC, and since our lower limit of quantification was 0.5 ng/mL anyone with a concentration ≥0.5 ng/mL was considered to have exceeded the “zero-tolerance” limit. Baseline concentrations of THC (and related compounds) were quantified prior to participants smoking cannabis. In this paper, we define baseline concentrations as the concentration of THC prior to initiation of smoking cannabis on the study day.

Details of the driving simulation along with how the composite drive score was calculated have been described previously (3). In short, the driving simulations were 25 min in length and recorded key variables such as standard deviation of lateral position, variability of speed, car following, and number of correct divided attention tasks. The validity of standard deviation of lateral position and car following tasks in detecting declines in performance relating to cannabis and other substances has been widely reported (20–26). The composite drive score normalized these variables based on presmoking driving simulation performance of all participants (3).

### Statistical Analysis

A paired *t*-test was used to test changes in THC concentration from baseline to 5 hours after smoking. A 2-sample *t*-test was used to compare driving performance between participants with baseline THC concentrations that exceeded per se and zero-tolerance cutpoints with participants who were below the cutpoints. Two-sided tests with significance level of 0.05 were used. For the Supplemental Tables evaluating the effect of participant demographics on baseline concentrations of THC, tests are independent samples *t*-test (age, BMI, days used), the Fisher exact test (sex, current use), or Wilcoxon rank sum test.

## Results

**Fig. 1.**
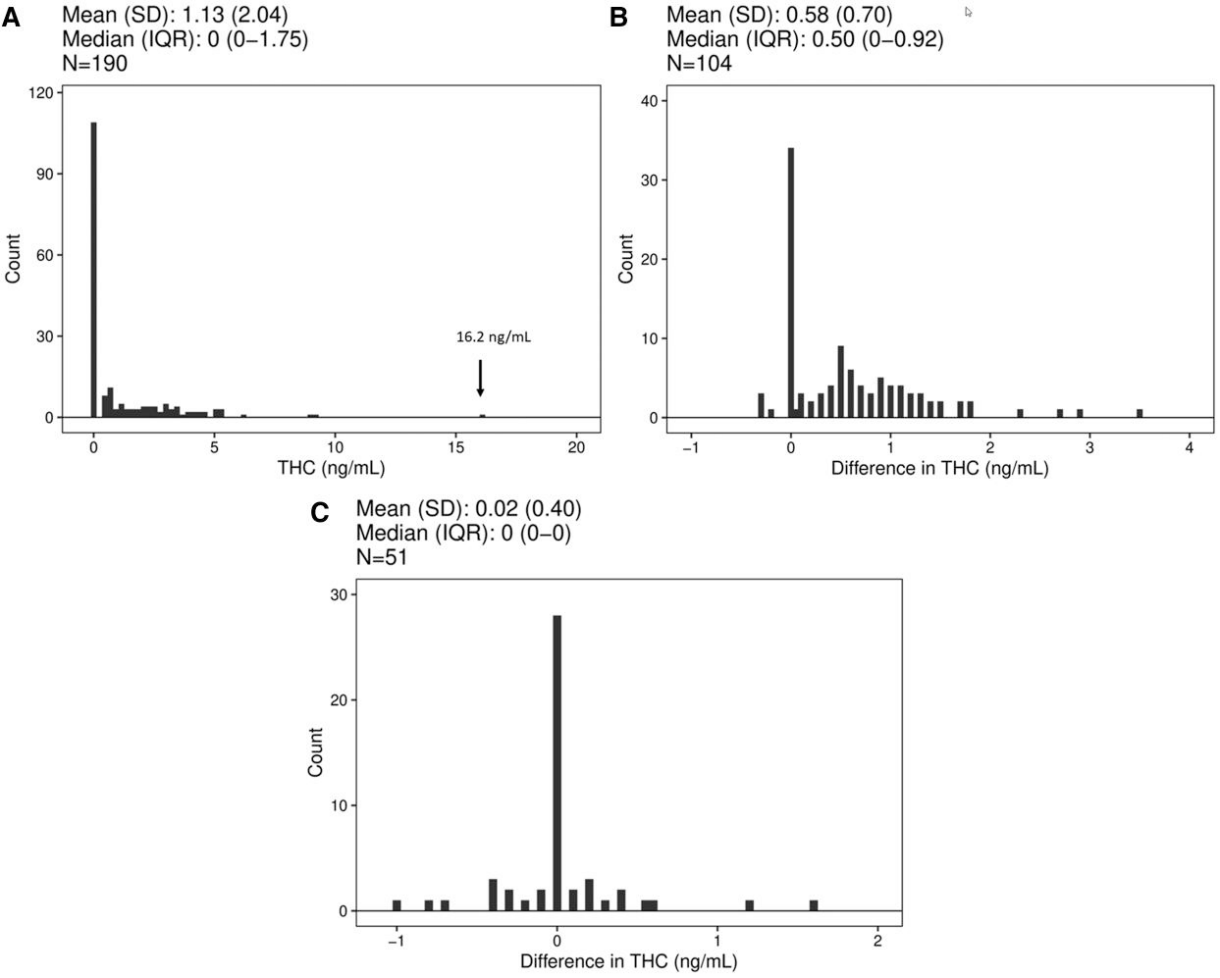
Distributions of THC concentrations. (A), The distribution of whole blood baseline THC concentrations (ng/mL) in 190 participants who were asked to abstain from using cannabis for 48 h prior to phlebotomy. The highest concentration of THC was 16.2 ng/mL. The median THC concentration at baseline was less than the lower limit of quantification (0.5 ng/mL); (B), The distribution of the difference in THC concentrations between the final blood draw (5 h postsmoking) and the baseline blood drawn (presmoking) for participants in the active drug arms of the study (*N* = 104). A 1-sample *t*-test comparing the mean change to zero was significant (*P* < 0.001), with a median (IQR) of 0.5 (0–0.92) ng/mL; (C), The difference between the final concentration and the baseline concentrations of THC in the placebo group (*N* = 51). A 1-sample *t*-test comparing the mean change to zero was not significant (*P* = 0.69), with a median (IQR) of 0.0 (0.0–0.0) ng/mL.

Participants self-reported that the median [interquartile range (IQR)] time since they last used cannabis was 3 (3–6) days. Figure 1A shows a histogram and range of the baseline blood concentrations of THC in all participants prior to the smoking session. The mean (±standard deviation) baseline THC concentration was 1.1 (*+/−* 2.0) ng/mL. The median (IQR) THC baseline concentration was 0 (0–1.8) ng/mL. The range of baseline THC concentrations was from 0 to 16.2 ng/mL. When considering per se cutpoints of 5 and 2 ng/mL, there were a total of 10 (5.3%) and 45 participants (24%) who exceeded these thresholds, respectively. Forty-three percent of the participants exceeded the zero-tolerance cutpoint at baseline. The baseline concentrations of other cannabinoids are shown in Table 1. A comparison of demographic characteristics based on the baseline THC blood concentration groups for the 3 cutpoints (5, 2, and 0.5 ng/mL) showed no statistically significant differences between the groups for age, sex, and BMI. All 3 cutpoints were associated with cannabis use variables. Specifically, people with higher THC concentrations were more likely to use cannabis more frequently, more recently, and in greater amounts (Supplemental Tables 1–3). Inclusion of predictors not correlated with the cutpoints (age, sex, BMI) did not change the effect of THC concentration on composite drive score, which remained not significant for all cutpoints.

**Table 1.** Median (interquartile range) baseline whole blood concentrations of cannabinoids (*N* = 190 participants).

Compound	Median (IQR); maximum ng/mL
THC	0 (0, 1.75); 16.2
CBN	0 (0, 0); 0.5
CBD	0 (0, 0); 2.5
11-OH-THC	0 (0, 0); 9.3
THCCOOH	3.25 (0, 15.2); 133
THCCOOH-Gluc	8.10 (0, 45); 571
CBG	0 (0, 0); 0
THC-V	0 (0, 0); 0

11-OH-THC, 11-hydroxy-THC; CBN, cannabinol; CBD, cannabidiol; CBG, cannabigerol; THC-V, tetrahydrocannabivarin.

To examine how baseline concentrations of THC compared to the final time point of the study, which was about 5 h after initiation of smoking, we subtracted the baseline THC concentrations from the final blood draw for participants not in the placebo arm (Fig. 1B). The concentration of THC was higher at the end of the study compared with baseline (*t* = 8.35, *df* = 103, *P* < 0.001) for the active drug cohort, but these differences were small with a median (IQR) change of 0.5 (0 to 0.9) ng/mL. The mode for this difference was 0 ng/mL, and 38 participants had a difference of 0 or less. Thirty-seven percent of participants’ blood THC concentrations had returned to baseline 5 h after smoking a cannabis cigarette containing 5.9% or 13.4% THC.

Figure 1C shows the difference between the final blood draw (about 5 h after smoking) and baseline concentrations for the placebo group, which had a median (IQR) value of 0 (0–0) ng/mL. This demonstrates that in the absence of smoking active cannabis, blood concentrations do not change appreciably over a 5 h time frame in a population of people who use cannabis. It should be noted that the lower limit of quantification of the analytical method is 0.5 ng/mL with accuracy of ±20% at this concentration (19).

**Fig. 2.**
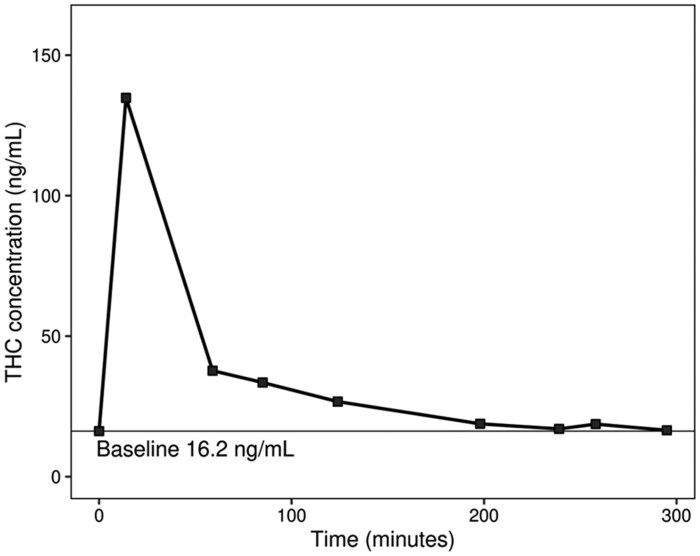
Kinetic profile of blood THC in the participant who had a baseline THC concentration of 16.2 ng/mL. This participant smoked 580 mg of a cannabis cigarette that contained 5.9% THC (34.2 mg of THC). Fourteen minutes after smoking, her blood THC concentration peaked at 135 ng/mL and by the end of the study (4 h 55 min after smoking) asymptotically approached her baseline concentration of 16.2 ng/mL.

One participant had a baseline THC concentration of 16.2 ng/mL. We were concerned that perhaps this participant used cannabis the day of the study given her high baseline concentration so we examined concentrations of metabolites and her kinetic profile more carefully. This participant also had the highest baseline concentrations of 11-hydroxy-THC, (±)-11-nor-9-carboxy-Δ9-THC (THCCOOH), and (+)-11-nor-Δ9-THC-9-carboxylic acid glucuronide (THCCOOH-gluc), which were 9.3, 133, and 571 ng/mL, respectively (Table 1). The kinetic profile of this participant, who was randomized into the low THC content group (700 mg of 5.9% THC), is shown in Fig. 2. Fourteen minutes after starting to smoke, this participant reached a peak THC concentration of 135 ng/mL. This participant’s THC concentration at the end of the study (5 h post smoking) was 16.5 ng/mL. Based on the difference between the pre- and postsmoking weight of the joint, this participant was exposed to 34.2 mg of THC. Other relevant characteristics of this participant were that she was 39 years old, female, self-reported using 0.5 g of cannabis 27 out of the last 30 days, and had a body mass index of 35.4. The participant self-reported as abstaining from cannabis for 2.5 days prior to the study. Her baseline (presmoking) performance on the driving simulator was slightly better than the mean for all participants. Kinetic profiles of other participants have been published previously (27, 28).

**Fig. 3.**
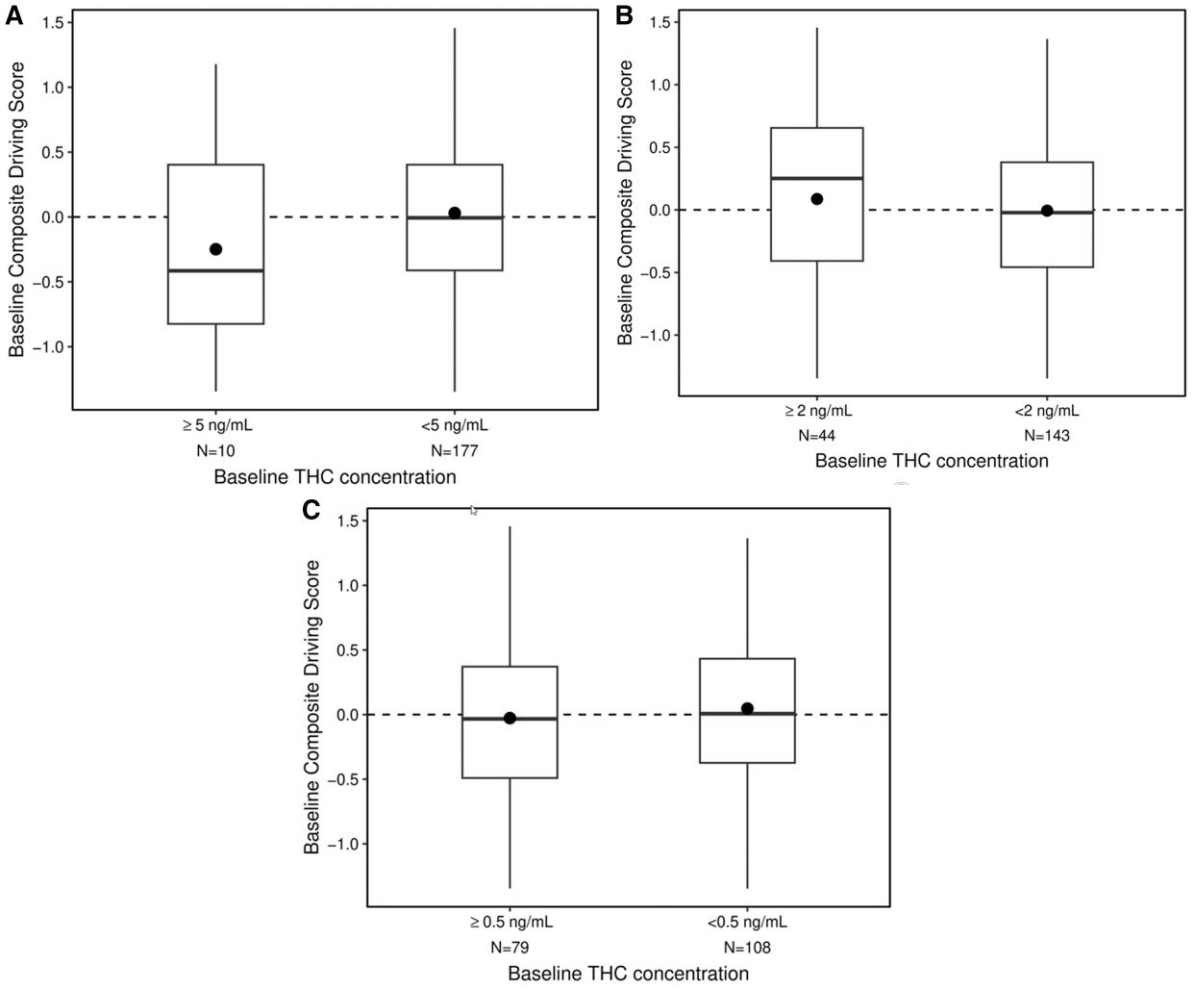
Baseline blood THC concentrations and performance on the driving simulator for participants who exceeded zero-tolerance and per se cutpoints with those who were below these cutpoints. (A), Baseline driving performance in participants with initial THC concentrations ≥5 ng/mL (*N* = 10) compared with participants who had <5 ng/mL (*N* = 177), *P* = 0.14; (B), Baseline driving performance in participants with initial THC concentrations ≥2 ng/mL (*N* = 44) compared with participants who had <2 ng/mL (*N* = 143) *P* = 0.36; (C), Baseline driving performance in participants with initial THC concentrations ≥0.5 ng/mL (*N* = 79) compared with participants who had <0.5 ng/mL (*N* = 108) *P* = 0.40. Solid bars indicate median values, dots are mean values, boxes represent the inner quartile ranges, and the whiskers show the range of observed values.

Using the composite drive score, we compared the baseline driving performance of participants with blood THC concentrations ≥5 and 2 ng/mL relative to participants with blood concentrations lower than these cutpoints. We did a similar analysis with participants above and below the zero-tolerance cutpoint (THC ≥0.5 ng/mL). Figure 3A shows the distributions of the composite drive scores for participants with baseline THC concentrations <5 ng/mL relative to those with baseline concentrations ≥5 ng/mL. Both of these distributions have a similar range of drive scores and were not significantly different (*t* = 1.47, *df* = 185, *P* = 0.14). Figure 3B shows a similar plot for participants with baseline THC <2 ng/mL and ≥2 ng/mL. Like the 5 ng/mL cutpoint, there were no differences in driving performance between participants above or below the 2 ng/mL cutpoint (*t* = −0.92, *df* = 185, *P* = 0.36). Figure 3C shows the comparison of driving scores between participants who had THC concentrations that were not quantifiable (<0.5 ng/mL) with those who exceeded zero tolerance (≥0.5 ng/mL). Like the other cutpoints, there were no differences in driving performance between participants above or below the zero-tolerance cutpoint (*t* = 0.85, *df* = 185, *P* = 0.40).

## Discussion

The results of this study are important for several reasons. We show that almost one-quarter of the population that regularly uses cannabis have baseline concentrations of THC that exceed the 2 ng/mL per se cutpoint and 5% exceed the 5 ng/mL per se cutpoint, while 43% of our participants exceeded the zero-tolerance cutpoint (0.5 ng/mL). Our data argue that the concentrations we measured at baseline likely reflect steady state THC concentrations in this population, several days after last use. We demonstrate that the baseline concentrations are not a result of recent exposure because the concentrations of THC at the end of the study are not significantly different from the baseline concentrations for the placebo group and were small (median difference of 0.5 ng/mL) for the active drug group. If participants had recently used (e.g., consumption the morning of the study) we would expect to see a difference between baseline concentrations and the final THC concentration. We also show, using quantitative data from the driving simulator, that participants who exceeded the zero-tolerance and per se cutpoints (2 and 5 ng/mL) performed in a similar manner as those below these arbitrary values. These results add to a growing body of evidence that per se THC blood statutes lack scientific credibility as prima facia evidence of impairment (9–12, 17, 27, 28).

Baseline concentrations of THC and metabolites in people who regularly use cannabis are difficult to determine because it is hard to control for time since last use. Desrosiers et al. measured baseline THC and related metabolites in a cohort (*N* = 25) that was in a secure research unit. Participants presented to the research unit 20 h before the baseline blood concentration was measured. Twenty hours after being in the restricted unit, the highest baseline concentration of THC measured was 8 ng/mL (29). In our larger study (*N* = 190) we had 3 participants with concentrations >8 ng/mL (9.0, 9.2 and 16.2 ng/mL). We present evidence that the baseline concentration of 16.2 ng/mL likely reflected this participant’s true baseline and not recent smoking (exposure the morning prior to the study period), because her final concentration measured (16.5 ng/mL) was within the error of the measurement of the analytical method (±20%) relative to her baseline concentration. If she had consumed cannabis the morning of the study we would have expected her baseline THC concentration to exceed the value at the end of the study. The fact that this participant had the highest concentration of THC metabolites and other cannabinoids as shown in Table 1, also supports our contention that the 16.2 ng/mL THC concentration measured reflects her true baseline.

Bergamaschi et al. also reported on baseline concentrations of THC and metabolites in 30 participants who resided in a secure research unit (17). On admission to the secure research area the median (range) of THC concentrations was 1.4 (0.3–6.3) ng/mL. After being in the secure unit for 1 day the highest measured THC concentration was 2.9 ng/mL and the highest THCCOOH concentration was 93.4 ng/mL (17). In our study, the highest THC concentration measured at baseline was 16.2 ng/mL and this participant had baseline concentrations of 133 ng/mL for THCOOH and 571 ng/mL for THCCOOH-glucuronide. It should be noted that Bergamaschi used a hydrolysis step to cleave THCCOOH-glucuronides (30), so the resultant THCCOOH represents both the free THCCOOH and the glucuronidated form. We did not include a hydrolysis step in our analytical method, so we report both THCCOOH and THCOOH-glucuronide. The initial concentration of 16.2 ng/mL of THC in our participant seems consistent with a baseline value since her total THCCOOH concentration is also much higher than that reported in the Bergamaschi study. While both THCCOOH and THCOOH-glucuronide are inactive metabolites, the very high concentrations we measured support that this participant was a heavy user of cannabis and are consistent with her elevated baseline (16.2 ng/mL) THC concentration.

Wurtz et al. reported that baseline blood concentrations exceeded the 5 ng/mL cutpoint of THC in 16 of 30 (53%) participants following a 12-hour period of abstinence and in the absence of impairment (both self-reported) (10). The median baseline THC blood concentration was 6.4 ng/mL, with the highest baseline concentration in the 80 ng/mL range (10). Most participants in the Wurtz study were described as chronic daily users. In our study we had 10/190 (5%) exceeding the 5 ng/mL cutpoint, but our participants had a more varied use history and a longer period of abstinence compared with the Wurtz report.

Odell et al. reported that the average blood THC concentration in a group (*N* = 21) of people who use cannabis was 4 ng/mL 32 hours after being admitted to a detoxification unit (31). These authors showed that concentrations of THC in blood varied from 1 to 13 ng/mL when people who use cannabis had been in the unit between 19 and 61 h. These results are similar to what we report for our participants who abstained for at least 48 h. Odell found that one participant had blood concentrations of THC that exceeded 5 ng/mL for 5 days, despite being in a detoxification unit. Odell also shows that several days after last use, the concentration of THC in blood fluctuates at low levels, generally ±1 or 2 ng/mL. The data we show in Fig. 1C, where the difference between the final THC concentration and the baseline concentration is not always 0 for the placebo group, likely represents what Odell observed. Odell postulated that small variations in the concentration of THC and metabolites at the later stages of excretion probably represents variable release of THC from body stores. Karshner et al. also reported similar findings where 89% of participants who were in a secure research unit had at least one specimen with increased THC concentrations compared to the previous day (32). As was suggested by Odell (31), the baseline blood THC concentrations we present are higher than previously reported, making it important to rethink how these concentrations are interpreted in a forensic setting.

The method for determining baseline or steady state concentrations of THC in blood is important especially considering per se driving under the influence statutes. Figure 2 shows that our participant with a baseline THC blood concentration of 16.2 ng/mL has a clear rise in concentration following the smoking session. Four hours after smoking, her blood concentrations asymptotically approach her initial baseline concentration. In states that have legal cannabis, it is very important that people who use cannabis are informed about the potential legal risks they face even when they have not used cannabis for several days or longer. Her baseline THC concentration greatly exceeds the zero-tolerance and per se cutpoints used by many states. Indeed, nearly half of our study population exceeds the zero-tolerance cutpoint and are at risk of the potentially serious consequences of a driving under the influence of drugs conviction when it has been several days since they last used cannabis. Our study adds important data to the literature showing that participants with elevated baseline concentrations of THC do no worse on a driving simulator compared with participants who are below per se cutpoints.

This study has several limitations. A primary limitation is that driving performance was gauged using a simulator that does not capture all of the variables encountered on roadways. However, the sensitivity and validity of the driving simulation has been assessed in a number of our publications (3, 33). In addition to the paper demonstrating that driving simulator performance was sensitive to THC exposure (3), we also found that across the entire sample, simulator performance was significantly (*P* < 0.001) associated with the total number of clues on field sobriety tests performed by certified drug recognition experts who were actively practicing in the field (33), providing additional external validation of the simulations. We have also shown that the current simulations are sensitive to alcohol impairment (34) and that prior iterations of the simulator relate to on-road driving evaluations conducted by certified evaluators (35). We also did not have a control group consisting of drivers who did not use cannabis. In addition, we did not sequester participants in a controlled facility for 2 days prior to the initiation of the study day to ensure they had not recently used cannabis. We relied on oral fluid testing and self-reporting to help ensure participants did not use the morning of the study. The kinetic data presented here also supports our intention of having participants abstain for 2 days prior to the beginning of the study.

More work needs to be done to address how to best identify drivers who are under the influence of cannabis and are unsafe to drive. A brief editorial highlights many of the challenges faced when developing a reliable test of cannabis impairment (36). At present, the best protocol is a combination of observations in the field and toxicology testing. We recognize that the current state of the art is lacking and have made recommendations on pathways for improvement (37). We feel that an essential component of improving highway safety is collaborations between law enforcement and the scientific community to develop standards that are unbiased and potentially lifesaving.

## Supplemental Material

Supplemental material is available at *Clinical Chemistry* online.

## Supplementary Material

hvaf121_Supplementary_Data

## Nonstandard Abbreviations

THC, delta-9-tetrahydrocannabinol; IQR, interquartile range; THCCOOH, (±)-11-nor-9-carboxy-Δ9-THC; THCCOOH-gluc, (+)-11-nor-Δ9-THC-9-carboxylic acid glucuronide.
