# Supplementary material for: Per Se Driving Under the Influence of Cannabis Statutes and Blood Delta-9-Tetrahydrocannabinol Concentrations following Short-Term Cannabis Abstinence
Source: Clin Chem. 2025 Nov 12;71(12):1225–33. doi: 10.1093/clinchem/hvaf121 (PMC12670588; doi:10.1093/clinchem/hvaf121)
Supplement: hvaf121_Supplementary_Data [file hvaf121_supplementary_data.docx]

Supplemental Table 1. Comparison of demographics of subjects with baseline concentrations of THC greater than or equal to 5 ng/mL with participants who had baseline THC concentration less than 5 ng/mL.

|  | THC < 5 ng/mL | THC >= 5 ng/mL | Test^1^: statistic (DF), p |
| --- | --- | --- | --- |
| Age, mean (SD) | 30 (8.2) | 32 (9.7) | t=-0.81 (188), 0.421 |
| Male sex, n (%) | 113 (62.8%) | 4 (40.0%) | 0.187 |
| BMI (kg/m^2), mean (SD) | 26.3 (5.3) | 24.5 (6.0) | t=1.05 (188), 0.294 |
| Cannabis Use |  |  |  |
| Current use < 4 times per week, n (%) | 97 (53.9%) | 1 (10.0%) | 0.008 |
| Days used in past 30 days, mean (SD) | 16.1 (9.7) | 26.8 (2.6) | t=-9.68 (28.1), <0.001 |
| Amount used in past 30 days (g/day), median (IQR) | 0.50 (0.25, 1.00) | 1.00 (0.81, 1.41) | W=1194.5, 0.047 |
| Days since last use, median (IQR) | 4.0 (3.0, 5.0) | 2.5 (2.5, 3.0) | W=268.5, 0.002 |

Notes: ^1^Tests are independent samples t-test (age, BMI, days used), Fisher’s exact test (sex, current use), or Wilcoxon rank sum test. Fisher’s exact test does not have a statistic.

Supplemental Table 2. Comparison of demographics of subjects with baseline concentrations of THC greater than or equal to 2 ng/mL with participants who had baseline THC concentration less than 2 ng/mL.

|  | THC < 2 ng/mL | THC >= 2 ng/mL | Test^1^: statistic (DF), p |
| --- | --- | --- | --- |
| Age, mean (SD) | 29 (8.0) | 32 (9.1) | t=-1.77 (188), 0.078 |
| Male sex, n (%) | 91 (62.8%) | 26 (57.8%) | χ^2^=0.36 (1), 0.548 |
| BMI (kg/m^2), mean (SD) | 26.5 (5.5) | 25.4 (4.9) | t=1.15 (188), 0.253 |
| Cannabis Use |  |  |  |
| Current use < 4 times per week, n (%) | 92 (63.4%) | 6 (13.3%) | χ^2^=34.5 (1), <0.001 |
| Days used in past 30 days, mean (SD) | 14.2 (9.3) | 24.1 (7.0) | t=-6.51 (181), <0.001 |
| Amount used in past 30 days (g/day), median (IQR) | 0.50 (0.25, 1.00) | 1.00 (0.55, 1.50) | W=4488.5, <0.001 |
| Days since last use, median (IQR) | 4.0 (3.0, 6.0) | 3.0 (2.5, 3.0) | W=1122.5, <0.001 |

Notes: ^1^Tests are independent samples t-test (age, BMI, days used), chi-square test (sex, current use), or Wilcoxon rank sum test.

Supplemental Table 3. Comparison of demographics of subjects with baseline concentrations of THC greater than or equal to 0.5 ng/mL with participants who had baseline THC concentration less than 0.5 ng/mL.

|  | THC < 0.5 ng/mL | THC >= 0.5 ng/mL | Test^1^: statistic (DF), p |
| --- | --- | --- | --- |
| Age, mean (SD) | 30 (8.3) | 30 (8.3) | t=-0.51 (188), 0.609 |
| Male sex, n (%) | 69 (63.3%) | 48 (59.3%) | χ^2^=0.32 (1), 0.571 |
| BMI (kg/m^2), mean (SD) | 26.7 (5.6) | 25.7 (5.0) | t=1.28 (188), 0.201 |
| Cannabis Use |  |  |  |
| Current use < 4 times per week, n (%) | 81 (74.3%) | 17 (21.0%) | χ^2^=52.9 (1), <0.001 |
| Days used in past 30 days, mean (SD) | 11.6 (8.3) | 23.0 (7.5) | t=-9.62 (181), <0.001 |
| Amount used in past 30 days (g/day), median (IQR) | 0.45 (0.25, 0.88) | 1.00 (0.50, 1.45) | W=5875, p<0.001 |
| Days since last use, median (IQR) | 4.0 (3.0, 7.0) | 3.0 (2.5, 4.0) | W=1577.5, <0.001 |

Notes: ^1^Tests are independent samples t-test (age, BMI, days used), chi-square test (sex, current use), or Wilcoxon rank sum test.
